# Radical Cure of Congenital Inguinal Hernia

**Published:** 1863-11

**Authors:** David W. Cheever


### Selections.

### RADICAL CURE OF CONGENITAL INGUINAL HERNIA.

By DAVID W. CHEEVER, M.D.

[Read before the Boston Society for Medical Improvement, Sept. 28th, 1863, and communicated for the Boston Medical and Surgical Journal.]

About six months ago, I operated on three cases of congenital inguinal hernia, with a view to attempt a radical cure. All were boys of from eight to twelve years of age. One case failed at the outset, ulceration having taken the place of the adhesive inflammation which was hoped for. The other two succeeded; and are thus far, six months after the operation, well. I should have preferred to wait until a year had elapsed before bringing these cases to the notice of the Society; but finding that one of the cases had moved away from the city, and fearing to lose sight of the other, I have brought him here to-night, to show the result of Wood's operation, and will exhibit him to the Society. Before doing so, it may be best to read a short account of the operations. The boy who has moved away was operated on by Gerdy's method; the other, by that of Mr. John Wood, of London, which consists in placing subcutaneous sutures around the inguinal canal.

CASE I.—Daniel S——, a healthy boy, 8 years of age, has a congenital ingenital hernia on the right side, as large as a hen's egg, when in the scrotum. The ring admits the forefinger with ease. The cord, testis, and spermatic plexus of veins are healthy. He has worn a truss, but latterly has been unable to keep the hernia up with it, and has left it off.

March 21st.—The bowels having been cleared, and the bladder emptied, he was etherized, placed on his back, the hernia reduced, and kept up by the finger of an assistant pressing over internal ring. The skin of the scrotum was invaginated into the the inguinal canal, and with the cord lying beneath the back of the finger, the inner pillar of the aponeurosis of the *external oblique* muscle and the conjoined tendon of the *internal oblique and transversalis* were raised upon the tip of the finger, about half an inch above the *pubes*. A curved needle armed with a silver suture was now entered over the tip of the finger from above the *pubes*, carried through the conjoined tendon, inner pillar and invaginated scrotum, and thence out below the *pubes*, where the invaginating finger was first entered. The needle was now detached, and then, threaded anew with another wire, it was entered from below, passed by the outer side of the finger through Poupart's ligament, and thence across the inguinal canal, emerging at the same point above the *pubes*, where the first suture entered. The four ends of the silver suture were then passed through two holes of a large button, and clamped pretty tightly over it. The boy now vomited and strained violently from the ether, but the hernia did not come down. He was now put to bed on his back, and an opiate administered. He remained strictly in the horizontal posture, and the wound was kept wet with cold water. With the exception of some pain and tenderness over the abdomen, accompanied with but little fever, everything went on well for three days.

March 24th.—He was given some castor oil. In the night he got up unperceived, walked to the water-closet, had a large evacuation, and sat and strained a long while. Severe orchitis now supervened, which ran a course of about a week, and then gradually subsided. It was treated with cold applications and opium. There was a good deal of purulent discharge around the sutures, and a sinus opened above the *pubes*. But the sutures held on; there was no descent of the intestine, and the testicle passed out of the acute stage. Three weeks and a-half after the operation the sutures were removed. In one week more the sinuses were closed; the hernia remained up, and the scrotum well invaginated. He was now, one month after the operation, allowed to get up, and walk about the room. No truss was applied.

May 23d, *one month* after getting up, and two months after the operation, the scrotum remained firmly invaginated; the testicle painless, and about one-third larger than the other; the inguinal canal filled with a dense deposit of lymph; the hernia up, and no bulging. I have seen this boy from time to time until very recently, and he has remained perfectly relieved; such is his state six months after the operation. I have very little fear of his hernia ever returning. He has worn no truss. He was kept in bed longer than the next case, which was let up too soon.

Case II.—William M——, 12 years old, has a congenital inguinal hernia on the right side. The ring is large, admitting the thumb without difficulty. Testicle, cord, and veins healthy; otherwise strong and active. Has worn different trusses that were made for him, but cannot keep it up with them. The difficulty is increasing as he grows.

April 13th.—After being etherized, and the hernia reduced and held up as before, an incision about three-fourths of an inch long was made through the skin of the scrotum of the right side, at its lower part. The fascia of the scrotum was now dissected subcutaneously from the skin, to which it adheres only by loose cellular tissue, for a space of about an inch and a-half in diameter all round; or until the fascia could be invaginated into the inguinal canal, without puckering the skin. The forefinger of the right hand being placed over the cord, and invaginating the fascia of the scrotum as high up into the inguinal canal as possible, a curved needle, with the eye in the point, and set on a firm handle, was next carried up along the finger, and made to perforate the *conjoined tendon* and *inner pillar* near the *internal* ring. The skin over the point of the needle was then drawn a little inwards and upwards, and it was made to emerge, when it was threaded with a silver suture and withdrawn, leaving one end of the wire projecting above the *pubes*. Next the finger was turned downwards and outwards beneath Poupart's ligament, pressing the cord back out of the way. The needle was next made to perforate Poupart's ligament, from within outwards, and as near the central point between the anterior superior spine of the *ilium* and the spine of the *pubes* as possible, and then, by drawing the skin downwards and outwards, the point was brought out at the same hole where the first stitch emerged above the *pubes*. Here a loop of the suture was retained, and the needle again drawn back. The finger being now turned upwards and *inwards*, and the needle following it, it was made to pierce the inner pillar and triangular ligament at the edge of the *rectus*, and again brought out, for the third and last time, through the same puncture above the *pubes*. The needle was now detached, and withdrawn with the finger. There were now left out above the *pubes* two free ends of the wire suture, which had passed through the inner pillar, one near the internal ring, and the other near the edge of the *rectus*

muscle; and a loop, whose other end was encircling Poupart's ligament, at a point midway between the *pubes* and *ilium*. The loop and the free ends were now crossed, and brought through two holes of a button, and clamped firmly over it. Previous to this, however, it was found, on passing the finger into the inguinal canal, that the fascia of the scrotum was tightly drawn up into this cavity, that the cord and testicle were free, and that on drawing the wires, the sides of the inguinal canal were approximated to each other. As in the former case, vomiting now came on and failed to bring down the hernia, and the same treatment was adopted as before. There was no orchitis; very little pain; not a bad symptom; on the contrary, it was feared that he was not getting up inflammation enough for a cure. There was a pretty free suppuration around the stitches, and through the incision in the scrotum. The sutures were removed in two weeks and a-half, and in four days more the wounds had closed. He was now allowed to sit up and move about the chamber. The hernia remained up, and there was some induration along the inguinal canal.

May 13th.—About one fortnight after getting up, a slight protrusion was noticed at the internal ring. Examination by the finger revealed the external ring reduced in size about one-half, with firm, sharp, and defined edges, showing it to be the result of actual approximation of its walls. There was much thickening of the scrotal fascia and cellular tissue over the ring. He was advised to wear a truss, with a weak spring and a flat pad, for some weeks. Within a few days after putting on the truss he left it off several hours while moving about, and the hernia did not come down. It has never come down since. He has worn the truss at first pretty continuously, then rarely, and for the last month not at all. Being an active boy he disliked the truss, and shirked putting it on when he could. During the last few weeks he has done heavy work, assisting in putting in coal, &c., without the truss, and with no bulging, or feeling of weakness in the groin. He considers himself well, and I think he is.*

These cases were operated on with silver wire,—the third case, which failed, with silk ligatures. Dr. Wood now gives the preference to the metallic suture. The instrument used

* When shown to the Society, the appearance of the parts was as follows:—A linear cicatrix on the right of the scrotum, and a slighter round one above the pubes. A little fulness along the course of the inguinal canal of the right side, and some induration. The testes alike, and the cord free. No puckering, or invagination of the skin of the scrotum. A small external abdominal ring can be felt. Not the slightest bubonocele—*six months after the operation.*

was not unlike an aneurism needle, with the eye in the point, and the latter somewhat sharpened.

Neither of these children were able to keep up their herniæ with a truss; or were benefited by one when worn, although fitted by the best makers of these instruments. It is extremely difficult to keep a truss well-fitted on a young, restless, and growing child; and we are inclined to think that the cases in which a hernia with a large ring, or a congenital one, is cured by a truss, are few in number, and the exception rather than the rule.

The opportunity which a subcutaneous dissection of the fascia over an enlarged ring affords for the anatomical study of the internal parts, is not the least interesting feature of this method of operating. Many things become very plain and palpable, which cannot be felt through the skin; such are the conjoined tendon, Poupart's ligament, the crural canal, the external iliac artery, &c.

In Gerdy's method, the skin covering the sac is invaginated and held in that position by a ligature thrust through the inter-columnar fascia and skin of the groin, till adhesion takes place at the point of ligature. The method proposed by Wützer, substitutes for the finger of the operator a wooden plug variously modified, with the intent to fill the canal and openings, and to stretch them so much as to set up adhesive inflammation all round the invaginated sac. The danger of peritonitis, which is regarded by many as a serious objection to any operation of this kind, may be considered as pretty nearly equal in all. But the results of numerous cases operated on seem to prove that the danger is by no means great, nor sufficient to deter the surgeon from endeavoring to cure this common deformity. "A much more awkward objection," says Mr. Wood, "is drawn from the inefficiency of these methods. In all the cases of Wützer's operation which have come under my observation, the result has been unsatisfactory; the rupture redescending on leaving off the truss." He goes on to enumerate the causes of failure as follows:—

The inefficiency of the steps taken to cause adhesion of the surfaces of the posterior fold of the invaginated sac together, and to the posterior wall of the canal. Into this fold, forming thus a secondary sac, the descent of a knuckle of intestine is imminent.

The action of the plug is to dilate the opening and the canal, instead of contracting them; the external ring and canal being left very patulous after Wützer's operation. The elastic reaction of the skin and the weight of the testis and scrotum tend, consequently, always to drag down the invaginated tissues.

In order to avoid these sources of failure, Mr. Wood thought better to proceed upon a principle directly opposite to that of dilatation, namely, that of *drawing together and compressing by ligature the abdominal opening and inguinal canal, so as to cause their sides to adhere together.* And he also thought best to give the operation *a subcutaneous character*, so as to reach to a higher point within the canal, and to lessen the bulk of the transplanted tissue. These two principles combined he claims to be new in the cure of hernia.

The results intended to be obtained may be briefly recapitulated as follows:—

The posterior and superior boundaries of the dilated canal are drawn forwards and downwards towards Poupart's ligament, and become united by inflammatory adhesion, in the area of pressure exercised by the ligatures, to the anterior and inferior boundaries. By the use of the two ligatures this takes place from the internal opening above, to the external ring below. The effect of this adhesion is to make the posterior wall act like a valve, excluding the bowel by closing against the anterior wall. This action is aided by the contraction of the cicatrized tissues, and increased by the subsequent downward traction of the testis and scrotum. In this way we have an assurance that the older the cure and the more the pressure, the greater the mechanical resistance and security against the return of the protrusion. The spermatic cord is embraced by the contracting tissues in the groove behind Poupart's ligament, which protects it from undue pressure.

In his work on Hernia, just published,* Mr. Wood gives the result of his operation in *sixty cases.* There was but one death, and that from pyæmia. There were forty-two cures; thus giving about 70 per cent of *successful* results. Many of these were children, and many, also, adults. A considerable number of the latter worked as sailors, coal-heavers, and dock-laborers without trusses, after the operation; and they were kept under observation during a period of a year, or more. It is reasonable to consider the danger of the operation less in a hernia the result of strain, than in a congenital one; for in the latter case we necessarily traverse the peritonial sac, and in the former we may not. This operation has also the advantage of render-

* On Rupture, Inguinal, Crural, and Umbilical; the Anatomy, Pathology, Diagnosis, Cause, and Prevention; with New Methods of effecting a Radical and Permanent Cure. By John Wood, F.R.C.S., &c. London, 1863.

ing a truss more efficient, even if it does not cure the hernia. For it leaves the rings smaller and the walls of the inguinal canal nearer together than they were before.

Mr. Wood has introduced some modifications of his method since these operations were done. But the essential principle is the same; to close the inguinal canal and both rings by approximation, and inflammatory adhesion. And this certainly seems the most reasonable method of attempting a radical cure.

